# Exploring the limitations of biophysical propensity scales coupled with machine learning for protein sequence analysis

**DOI:** 10.1038/s41598-019-53324-w

**Published:** 2019-11-15

**Authors:** Daniele Raimondi, Gabriele Orlando, Wim F. Vranken, Yves Moreau

**Affiliations:** 1 ESAT-STADIUS, KU Leuven, 3001 Leuven, Belgium; 2 Interuniversity Institute of Bioinformatics in Brussels, ULB-VUB, 1050 Brussels, Belgium; 3 Structural Biology Brussels, Vrije Universiteit Brussel, Brussels, 1050 Belgium

**Keywords:** Sequence annotation, Machine learning, Protein sequence analyses

## Abstract

Machine learning (ML) is ubiquitous in bioinformatics, due to its versatility. One of the most crucial aspects to consider while training a ML model is to carefully select the optimal feature encoding for the problem at hand. Biophysical propensity scales are widely adopted in structural bioinformatics because they describe amino acids properties that are intuitively relevant for many structural and functional aspects of proteins, and are thus commonly used as input features for ML methods. In this paper we reproduce three classical structural bioinformatics prediction tasks to investigate the main assumptions about the use of propensity scales as input features for ML methods. We investigate their usefulness with different randomization experiments and we show that their effectiveness varies among the ML methods used and the tasks. We show that while linear methods are more dependent on the feature encoding, the specific biophysical meaning of the features is less relevant for non-linear methods. Moreover, we show that even among linear ML methods, the simpler one-hot encoding can surprisingly outperform the “biologically meaningful” scales. We also show that feature selection performed with non-linear ML methods may not be able to distinguish between randomized and “real” propensity scales by properly prioritizing to the latter. Finally, we show that learning problem-specific embeddings could be a simple, assumptions-free and optimal way to perform feature learning/engineering for structural bioinformatics tasks.

## Introduction

Machine Learning (ML) has become the Swiss army knife for solving many Bioinformatics problems and it has led to many successes in recent years. In particular, in structural Bioinformatics, where much effort is devoted to the prediction of functional and structural properties of proteins, researchers are constantly in need of efficient and meaningful ways to *encode* their knowledge about the amino acids composing the protein sequences into some ML-understandable numerical representation, commonly known as *feature vectors*. Selecting informative features is indeed crucial in ML since they convey the description of the objects under investigation, representing their most relevant characteristics. These values are generally organized in vectors representing points in a high-dimensional space, in which ML algorithms can learn decision boundaries for classification, regression or clustering.

For this reason, finding the optimal feature representation for each problem is a central issue for bioinformaticians, and improvements in both ML models and the features used have contributed to a great deal of positive results in the field, such as the prediction of protein Secondary Structure^1–3^, Relative Solvent Accessibility^4,5^, Contact Maps^6,7^, homology^8,9^, cysteine oxidation states^10,11^, early folding events^12^, the deleteriousness of variants^13–15^ and many more protein-related aspects.

The information contained in the protein sequences is crucial for the protein folding process and to allow the proteins perform their unique functions^16^. Therefore, many different features have been used to represent the amino acids composing the proteins under scrutiny, with among the most successful evolutionary information^1,2,5,17^ obtained from Multiple Sequence Aligments (MSAs) computed by methods such as HMMER^18^ or HHblits^19^. These MSAs are transformed into feature vectors by building sequence profiles or Position-Specific Scoring matrices^17^ from them. Features derived from MSAs have been shown to be helpful for many protein-related prediction tasks^5^, since they can describe the evolutionary history of the target proteins, thus providing hints on where functionally and structurally relevant parts are located, and possibly providing insights into the evolutionary constraints to which the protein family has been subjected. On the other hand, MSAs are computationally expensive, since they require queries through tens of millions of sequences, and are therefore not always a feasible option for predicting large scale problems. Moreover, they may bias a predictor towards well studied proteins, for which many more homologous sequences tend to be known^20^, or sometimes the goal of the study goes beyond the detection of an evolutionary signal.

The latter scenario involves many bioinformatics studies (*e.g*.^12,21–23^), and the most commonly adopted solution in these papers is to describe the amino acid residues in the protein sequence by values that characterize their physico-chemical behavior, such as their hydrophobicity, charge, volume, molecular weight or polarity^14^. The use of such *propensity scales*, which essentially identify *self-properties* of amino acids, provides an extremely simple and intuitive way to describe the amino acids within a protein. They are very popular in structural bioinformatics, with propensity scales being readily available through databases such as AAindex^24,25^, which contains 566 scales and more than 100 similarity matrices between amino acids. The two AAindex publications combined have more than 1000 citations, as evinced from Google Scholar, but this under-estimates the usage of propensity scales in this field because many studies reference the original papers that introduced the scale in the first place^14^. The use of scales in ML methods in Bioinformatics spans among different applications, goals of the studies and Impact Factor (IF) of the journal; a partial list of the most notable papers (with more than 100 citations) using them is the following:^14,21–23,26,27^. Other examples of papers with a number of references greater than the IF of the publishing journal are the following^28–33^:

In this paper we take a detailed look at the widespread use of propensity scales as features for the ML-based solution of structural bioinformatics problems. We performed different in-silico experiments reproducing typical bioinformatics tasks, with our results disputing the commonly accepted notion that the intuitively meaningful propensity scales are the most straightforward way to encode amino acid information for the purpose of prediction and inference with ML methods. Our conclusions are counter intuitive with respect to many approaches developed so far, since we show that on certain problems, the simpler ONEHOT encoding can outperform the scales, even if this encoding is the least informative possible with regard to the amino acid characteristics. Secondly, we show that, in particular when using non-linear ML methods, propensity scales become just an arbitrary way to provide a numeric description of residues (i.e. an embedding) and that extremely similar results are obtained after the “biological meaning” is removed with different types of randomizations. Finally, we show that feature selection may not be always able to distinguish between randomized and real scales and we thus suggest the use of gradient-descent-based learning of embeddings as a simple and assumptions-free way to learn optimal features to solve structural bioinformatics tasks.

## Materials and Methods

### Datasets

We used the SCRATCH-1D dataset^3^, to train and test Secondary Structure (SS) and Relative Solvent Accessibility (RSA) predictors used in our experiments. It contains 5492 proteins with less than 25% Sequence Identity (SI) between them. They have been annotated with 3-state SS and RSA assignments from DSSP. A residue is considered to be exposed to the solvent if it is above the 25% exposure threshold^3^. In each iteration of our SS and RSA experiments, we used 1500 randomly selected proteins for the training and 1000 for testing.

We used the PDBCYS dataset^10^ to train and test the cysteine oxidation (OXCYS) predictor. The PDBCYS dataset contains 1797 proteins with 3194 oxidised and 7619 reduced cysteines (considering only the ones involved in intra-chain disulfide bonds). We used BLASTCLUST to reduce the SI within the sequences in the dataset below 20%, obtaining a final set of 1743 proteins. In each iteration of our OXCYS experiment, we used 70% of these proteins as train set and the remaining 30% as test set. These sets are randomly selected at the beginning of each iteration of our simulations.

### Propensity scales

We extracted the propensity scales used in our experiments from the AAindex database, version 9^25^. Since many of these scales describe very similar biophysical properties or they have been just derived from slightly different experimental settings, there is a certain level of redundancy within the database. We thus decided to limit this redundancy and to consider only a sufficiently *diverse* set of scales. To do so, we filtered the total 566 scales by selecting a subset of scales sharing less than 0.6 of Pearson’s correlation between each other, obtaining a final pool of 85 scales with reduced redundancy. We refer to this subset as AAindexNR.

The simplest possible “scale” used to describe amino acid is the one-hot encoding, in which each amino acid is described by a 20 dimensional vector with 19 positions set to 0 and only the position identifying the specific amino acid type set to 1. This encoding scheme does not convey any information regarding the similarity between amino acids because they are all equidistant in their feature space. We refer to this encoding as ONEHOT.

### Sliding window

A common procedure in bioinformatics is to represent each residue in the sequence by considering a *window* of residues flanking it. For example, residue in position *i* could be represented by a window including the residues from position *i* − *w* to *i* + *w* + 1. The total length of this region (2 × *w* + 1) is called *window size* (WS). During our experiments we randomly sampled the window sizes (WS) from the following set of values: *WSET* = {3, 5, 7, 9, 11, 13, 15, 17, 19, 21, 23}.

### Machine learning algorithms

We ran our experiments with ML methods with different characteristics, such as Random Forest (RF), Multilayer Perceptron (MLP), Ridge Classifier (Ridge) and Linear SVM (LinSVC). All the implementations are provided by the scikit-learn library^34^, version 0.19.1. In all methods we used the default parameters (see Suppl. Material), except for the RF, which uses 50 trees and the MLP, which has 50 hidden units and the maximum number of epochs is set to 70.

### Evaluation metrics

To evaluate the cysteine oxidation states (OXCYS) and Relative Solvent Accessibility (RSA) prediction tasks, which are classical binary classification problems, we used the Area Under the ROC curve (AUC). To evaluate the Secondary Structure (SS), which is a three-class classification problem, we used the Matthews Correlation Coefficient (MCC). Effect sizes have been computed with Cohen’s *d*^35^. See Suppl. Mat. for more details.

### Randomization experiments on structural bioinformatics tasks

The design goal of our in-silico experiments is to test the behavior of different ML methods on some typical and representative structural bioinformatics tasks while adopting different feature encoding schemes and window sizes. To do so, we devised a fully automatic procedure to train and test the four ML methods *M* = {*RF*, *MLP*, *Ridge*, *LinSVC*} under varying conditions (sliding window size and selection of scales) for each bioinformatics task *T* = {*RSA*, *SS*, *OXCYS*}. For each task *T* and each method *M*, we repeated the training and testing experiments 500 times, randomizing the subset of scales and the window size used for every iteration *i*. Within each iteration *i*, we randomly selected a subset *S* containing 1 to 10 scales from AAindexNR and a specific WS from WSET. We limited the number of scales to 10, thus limiting the maximum number of dimensions in the feature vectors to 230 (corresponding to *S* = 10 and *WS* = 23) to reduce the risk of over-fitting and to limit the computational time of each iteration of the experiments.

During each iteration *i*, the train and test datasets are randomly selected, in a 70%-30% fashion for *T* = *OXCYS* and by using 1500 proteins for training and 1000 for testing in case *T* = {*RSA*, *SS*}. For each combination of (*i*, T, M, WS, S) we ran the following 5 train and test procedure, and stored the obtained results.

ONEHOT: to investigate the performance of a feature encoding scheme which is completely agnostic with respect to the similarities or differences between amino acids, we tested the classical ONEHOT-encoding (ONEHOT), randomizing the WS 100 times for each task *T* and ML method *M*. We refer to this experiment as ONEHOT in the plots.

- REAL: in this set of simulations we used the actual scales sampled from AAindexNR to reproduce their use within an prototypical structural bioinformatics pipeline. In each iteration we selected a subset S of scales from AAindexNR and a window size WS to build feature vectors for the training and test sets. This is called REAL in the violin plots showing the distributions of the scores, because it represents the real case of use of the scales in a bioinformatics pipeline.
- SHUFFLED: This is the first form of randomization of the scales that we tested. During each iteration of the simulation we randomly shuffled the values within each of the scales in S before computing the features with the corresponding WS. This is referred as SHUFFLED in the plots because the actual values of the scales S sampled from AAindexNR is preserved, but their correspondence to the amino acids is randomly shuffled.

RANDGEN: In this case, we actually disregarded the scales in *S* and we randomly generated an equal number of scales |*S*| by random sampling their values from uniform distributions with the same range of the original scales. This is called “RANDGEN” in the rest of the paper.

REAL RANDOM: Here we show how the ML inference within a pseudo-random scenario looks like, providing comparison with the other kinds of randomizations described above. In this simulation we described each residue in the proteins with a random vector with *WS* × |*S*| dimensions generated on the fly from a uniform distribution between −1 and 1.

## Results

Here we show the results obtained with our randomization experiments on 3 prototypical structural bioinformatics tasks. We briefly describe each task and we show how the classic use of biophysical propensity scales compares, in terms of prediction performance, to the simpler ONEHOT encoding and the two forms of randomization of the scales.

### Relative solvent accessibility prediction experiment

The goal of the protein Relative Solvent Accessibility (RSA) prediction task is to discriminate between residues that interact with the solvent, at the surface of the protein, and residues that are shielded from solvent because they are buried in the hydrophobic core of the protein. For simplicity, this problem is usually structured as a binary classification task in which Bioinformatics methods aim at predicting the exposed/buried state of each residue in the protein sequence. Residues with more than 25% of relative exposure to solvent belongs to the first class and all the others to the second^4^. For more details about this task, see^4,5^.

In our experiment we reproduced the classical RSA prediction pipeline, in which each residue in each protein is represented as a numeric feature vector computed by using the sliding window technique and the propensity scales from AAindexNR, or one of the randomized encodings described in Methods. We ran our experiments evaluating the performances of two non-linear ML methods (Multi-Layer Perceptron (MLP) and Random Forest (RF)) and two linear ones (Ridge Classifier (Ridge) and Linear Support Vector Classifier (LinSVC)).

**Figure 1:**
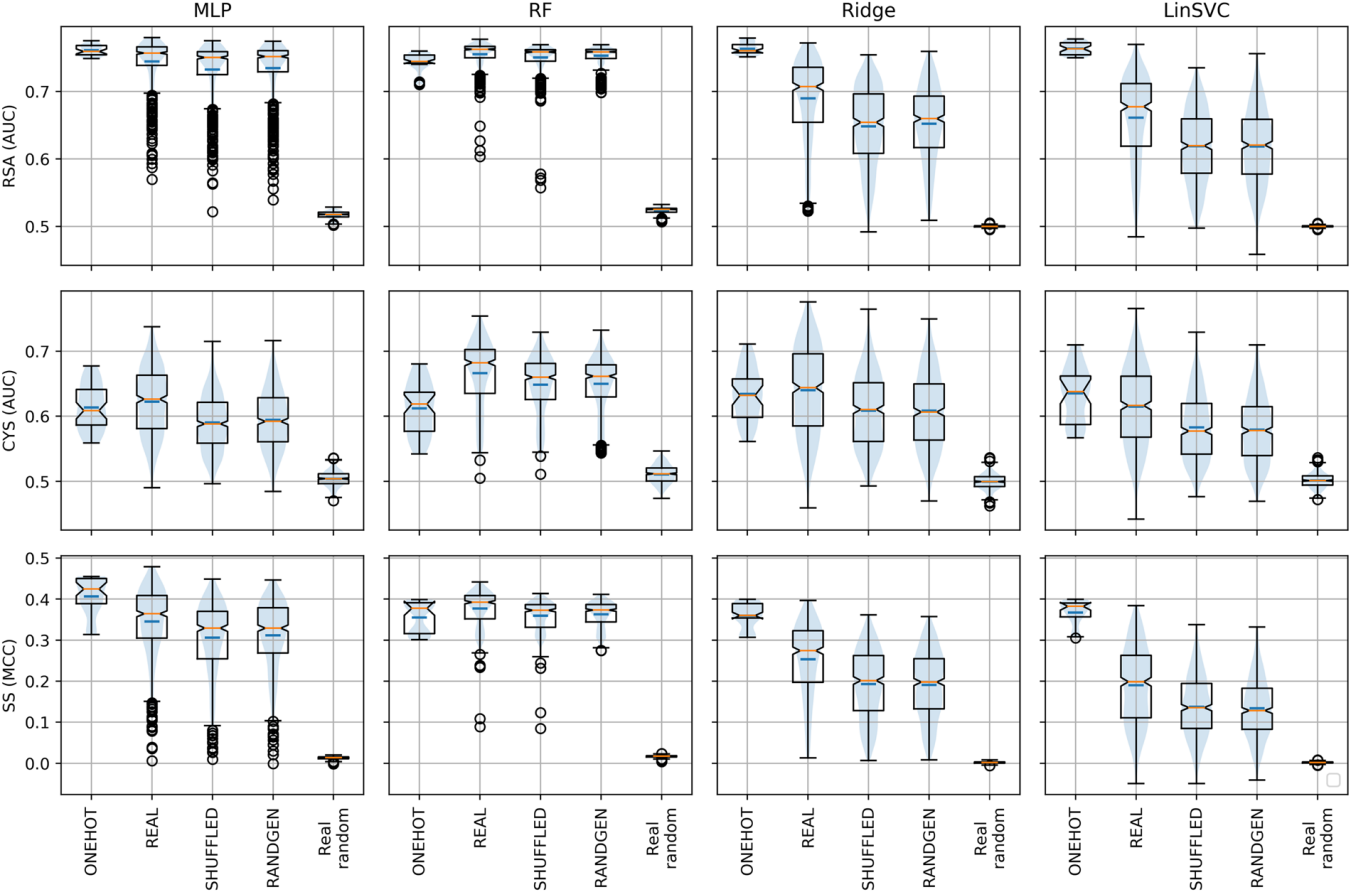
Results of the randomization experiments. This figure shows the results of the experiments on the 3 tasks tested in this study (Cysteine oxidation (CYS), Relative Solvent Accessibility (RSA) and Secondary Structure (SS) predictions). The plots show the performances obtained by 4 ML methods (Multilayer Perceptron (MLP), Random Forest (RF), Ridge classifier (Ridge) and Linear Support Vector Machine (LinSVC)) in terms of their AUC (MCC in the SS case). For each ML method and each task we ran five simulations, testing the scores obtained with different features encoding. For each combination of task and ML method, we tested the ONEHOT encoding (ONEHOT), randomly sampled propensity scales (REAL), randomly shuffled propensity scales (SHUFFLED), randomly generated scales (RANDGEN) and a true randomization of the vectors (see Methods for more details).

The first row of Fig. 1 shows the distribution of the Area Under the ROC curve (AUC) scores obtained by the four ML methods analysed (MLP, RF, Ridge, LinSVC) during the 500 iterations of our randomization experiment on the RSA task. On average, 47% of the samples in each iterations are positives, leading to a balanced prediction problem. Although the Wilcoxon ranksums p-values are always significant, we can see that in the case of non-linear methods such as MLP and RF, the SHUFFLED and RANDGEN scales give AUC scores that are very similar to the ones obtained with the REAL biophysical scales. To quantify the magnitude of the difference between the performance obtained by the REAL biophysical scales and the randomized ones, we then used the Cohen’s *d* (see Methods) to evaluate the effect size, measured in standard deviations (stds).

The observed effect sizes *d* are indeed quite modest, and the SHUFFLED and RANDGEN scales obtain results that are just 0.10–0.3 stds lower that the REAL distribution of AUC scores. This indicates that these non-linear methods do not get much benefit by describing the amino acids with the actual propensity scales extracted from AAindexNR instead of i) the shuffled version of the same scales or ii) randomly generated scales from an uniform distribution.

Moreover, the ONEHOT encoding, which is completely agnostic about similarities between aminoacids, and thus should convey less information than biophysically meaningful scales, consistently provides results as good as the ones obtained with REAL scales in the MLP case, but lower AUC (0.63 stds lower) in the RF case.

When we apply linear methods, which are less flexible in terms of the decision boundary that they can learn, they exhibit a higher dependence on the feature encoding compared to RF and MLP (see Fig. 1). This is apparent from the fact that the AUCs obtained by SHUFFLED and RANDGEN scales are lower than the ones obtained with the REAL scales (0.65–0.70 stds lower for Ridge and 0.73 stds lower for LinSVC).

Surprisingly, the ONEHOT encoding performs really well with these linear models. The AUC scores obtained by ONEHOT are respectively 1.25 stds higher for Ridge and *d* = 1.71 stds higher for LinSVC, with respect to the REAL scales. In particular, this encoding, which should intuitively convey less information, (i) outperforms all the feature encodings derived from AAindex propensity scales and (ii) performs on par with non-linear methods.

It is also important to notice that the number of dimensions in the feature vectors used by the ONEHOT experiments is comparable to the ones used in all the other experiments (60–230 features) and thus the ONEHOT encoding performances are not likely explained by the sheer number of dimensions (see Suppl. Fig. S1).

### Cysteine Oxidation prediction experiment

The second experiment we carried out relates to the discrimination between oxidised and reduced cysteines. Cysteines are an atypical amino acid because the thiol group in its sidechain can undergo oxidation^11^ to form a disulfide bond with the thiol group of another cysteine in the same (intra-chain disulfide bonds) or in another protein (inter-chain disulfide bonds). These strong covalent bonds can have structural, allosteric and catalytic roles in proteins^11^. The goal of the cysteine oxidation prediction task (OXCYS) is to train a classifier able to discriminate between cysteines that are present in the sequence in their reduced form with respect to cysteines forming a disulfide bond with another cysteine belonging to the same protein chain and it is thus formalized as a binary classification problem. Bioinformatics methods devoted to this task^10,11^ generally use the sliding window technique to extract evolutionary information in the form of sequence profiles derived from MSAs.

The second row of Fig. 1 shows the distributions of the AUC scores obtained by the four ML methods on the OXCYS task. We can see that this problem is more difficult to solve with respect to RSA, with generally lower AUC scores obtained. Among the non-linear methods, the RF generally outperforms MLP. The RF scores obtained with the SHUFFLED and RANDGEN scales are generally 0.34–0.36 stds lower than the AUCs obtained with the REAL scales, while the ONEHOT distribution is more than one std lower. The effect size of the differences between REAL and randomized scales in the MLP experiment are larger and range between 0.58–0.67 stds. For both methods, the best performing encoding is the one using REAL propensity scales. On average, 28% of the samples in each iteration are positives.

The linear methods (Ridge, LinSVC) show a behavior similar to the MLP, meaning that the REAL scales encoding is slightly preferable with respect to the other encodings, in particular in the Ridge case. The second most effective encoding is the ONEHOT. Overall, linear methods scores have generally higher variance and lower mean and medians with respect to the best performing non-linear method, the RF.

### Secondary structure prediction experiment

When folded, the protein chain forms energetically favorable local Secondary Structures (SS) elements, which are stabilized mainly by hydrogen bonds between the residues’ backbone atoms^1^. The prediction of which SS conformation is likely to be adopted by a residue in a protein is inherently a multi-class classification problem, and in literature different SS definitions have been proposed, from 8 to 3 separate SS classes^2^. In our experiments we adopt the 3-class SS definition, which involves alpha-helices (helices in which each turn comprises 3.6 residues), beta-strands (regions with extended backbone conformations participating in beta-sheets) and coils (regions which are not in the previously mentioned states). SS prediction is one of the first problems to be extensively addressed by bioinformatics, and over the years many methods have been developed. These algorithms generally predict by representing each residue with a sliding window technique^1^ or they iterate over the sequence using Recurrent Neural Networks^2,3^.

The last row of Fig. 1 shows the results of the experiments obtained on the SS task. Among the non-linear methods we can see that the REAL and randomized distributions provide similar results. In particular, for both RF and MLP, the MCC scores obtained by randomized scales are just 0.45–0.35 stds lower than the real AAindex scales. In the case of MLP, the ONEHOT encoding gives on average better scores than REAL scales (0.74 stds better), while ONEHOT gives poorer performance in the RF context (0.48 stds lower). Overall, non-linear methods perform better than linear ones in the SS prediction task. In the case of linear methods, ONEHOT encoding clearly dominates over the REAL scales, providing results 1.21 stds higher for Ridge and 1.95 stds higher for LinSVC. As is commonly observed for linear methods, the randomized scales tend to perform lower than the REAL scales, with effect sizes ranging between 0.63–0.72 stds lower.

### Factoring out the sheer feature size contribution

The size of the feature vectors used in ML practice is a very delicate parameter, because it is closely linked to the risk of obtaining inflated performances due to over-fitting. Indeed, the size of the feature vectors conditions the number of adaptive parameters in many ML algorithms, and thus influences the complexity of the final model.

To investigate how the sheer number of features used in our experiments affects the results shown so far, Suppl. Fig. S1 shows the AUC scores obtained in our experimental settings in function of the feature vectors’ sizes. The AUC scores, or MCC in the case of the SS prediction, improve with an increasing number of features used, regardless of the feature encoding. In particular, the SHUFFLED and RANDGEN scores are generally similar, while the use of REAL scales leads to higher means. Nonetheless, these means very often fall within the error bars of the scores obtained by the randomized experiments, which represent the standard deviation.

In the RSA and SS experiments, non-linear models tend to reach optimal or near-optimal scores with relatively small feature sizes (between 50 and 100 dimensions) and show little benefit when this number is increased. A striking point concerning linear models is that in the SS and RSA experiments, ONEHOT encoding consistently outperforms the other encodings, regardless of the number of features used. This is not the case for the CYS prediction, where ONEHOT performs poorly regardless of the ML method used. In the case of non-linear methods, ONEHOT is not very effective in the RF context, likely because its binary nature does not cope well with the binary decision trees learned by the Random Forest algorithm.

### Analysis of the best scores

Many bioinformatics papers (e.g.^22,23,26^) that use physico-chemical scales do not focus on the distributions of the AUC scores obtained while varying the parameters, but perform parameter optimization searches in order to identify the optimal combination of scales, window-sizes and ML parameters for the problem under scrutiny. Table 1 shows the highest score obtained for each ML method in each experiment. In the RSA case, the best results obtained among non-linear methods (MLP, RF) uses REAL scales, but they are just 0.65–1% higher than the best result obtained with randomized scales. For what concerns linear methods, where the gap between randomized and REAL scales is more pronounced (3–6%), the ONEHOT encoding slightly (1%) outperforms the other scales. Similar results are obtained with the other experiments. In the OXCYS task, using REAL scales gives a boost of around 3% of AUC with non-linear models and between 1% and 4% with linear ones. In the SS task, coupling REAL scales with non-linear ML methods gives a boost of 6.5–7% and 10–13% for linear models, even though the ONEHOT encoding gives the best performance in the latter case.

**Table 1:** Table showing the best AUC (MCC in the case of SS task) scores obtained in our simulations.

Task	Method	ONEHOT	REAL	SHUFFLED	RANDGEN
RSA	MLP	77.5	**78.0**	77.5	77.4
	RF	76.0	**77.7**	76.9	76.9
	Ridge	**77.9**	77.2	75.4	75.9
	LinSVC	**77.8**	77.0	73.5	75.6
CYS	MLP	67.7	**73.7**	71.5	71.6
	RF	68.0	**75.4**	72.9	73.2
	Ridge	71.1	**77.6**	76.5	74.9
	LinSVC	71.0	**76.5**	72.9	71.0
SS	MLP	45.5	**47.9**	44.9	44.6
	RF	39.9	**44.1**	41.4	41.2
	Ridge	**40.0**	39.7	36.1	35.7
	LinSVC	**40.0**	38.4	33.8	33.2

### Additional randomization experiments on the homology detection task

To highlight the fact that the behavior that we are analysing is not task-dependent, but is instead generated by the coupling of propensity scales as feature vectors in non-linear machine learning methods, we ran an additional randomization experiment on the homology detection task, using completely independent data from the datasets used so far.

In particular, we adapted WARP^9^, which is a recently published alignment-free method for global homology detection to take propensity scales as features describing the proteins under analysis, and we reproduced one of the validations performed in the original paper (see^9^ and Suppl. Mat. Section S7 for more details). From the AAindex scales we selected 85 non-redundant propensity scales (ensuring a pairwise Pearson’s *r* ≤ 0.6) and we ran 100 training and testing procedures of WARP on the PFAM dataset used in^9^, which contains 5245 homologous pairs of proteins (the positive cases) and 5245 non-homologous pairs of proteins (see Suppl. Mat. Section S7 for more details). During each iteration:

- we randomly selected 5 propensity scales from this pool, and we trained and tested WARP, computing the “REAL” AUC score;
- we then individually shuffled the same 5 scales, repeating the training and testing procedure and thus obtaining the AUC score for the “SHUFFLED” scales.

The plot showing the distribution of the REAL and AUC scores obtained during these 100 iterations are shown in Suppl. Fig. S4. The difference between the distributions obtained in this experiments are compatible to what has been shown for the SS, RSA and OXCYS tasks: while the REAL scales have a slightly higher mean AUC, the SHUFFLED scales provide very close scores, which are also significantly higher than the random performances.

Moreover, while developing a bioinformatics pipeline researchers are generally more interested in identifying the best performing set of parameters, instead of just analysing the distribution of the possible AUC scores. In Suppl. Table S1 we therefore compare the best AUC obtained by the REAL scales on the homology detection task (0.81) with the best AUC obtained by SHUFFLED scales (0.8). In this case, the benefit of using *biologically meaningful* propensity scales over randomly shuffled scales amounts to 1,25% of AUC and 9% of AUPRC (see Suppl. Table S1 and Suppl. Mat. Section S7 for more details).

### Real vs. Shuffled scales from a feature selection perspective

To further investigate the benefits of using the REAL biophysical propensity scales to solve structural bioinformatics problems with ML, we performed an additional experiment, involving feature selection over the RSA prediction task. We created a pool of scales composed by the 553 scales from AAindex plus the randomly shuffled version of the same 553 scales, obtaining a set of 1106 scales. From this pool we sampled, using a round robin approach, one REAL and one SHUFFLED scale at a time, adding them to a set *P*_*x*_ until more additions were not possible. In order to obtain meaningful results, we ensured that the scales added to *P*_*x*_ had limited redundancy with the ones already in *P*_*x*_. We regulated the redundancy by ensuring that each newly added scale had less than *x* = 0.4 or *x* = 0.6 of Pearson correlation with the others. We called *P*_0.4_ and *P*_0.6_ the two sets obtained in this way. When no more additions to *P*_*x*_ were possible, due to the redundancy constraints, we corrected the proportions of REAL and SHUFFLED scales in *P*_*x*_ obtaining an even number of REAL and SHUFFLED scales (69 REAL and 69 SHUFFLED for *P*_0.6_ and 16 and 16 for *P*_0.4_).

Starting from these non redundant pools of scales, we used the approximated Shapley value^36^ to evaluate the contribution of features derived from REAL and SHUFFLED scales towards the solution of the RSA problem. The Shapley value^37^ derives from cooperative game theory and is used to fairly assign to each player in a coalition a share of payoffs proportional to its contribution among the cooperating players. This method has already been used as feature selection algorithm^38,39^ and we adopted it here to test the hypothesis that features obtained from REAL propensity scales should be more relevant towards the solution of bioinformatics problems such as the RSA task. Considering the sets of scales used in the ML context as a coalition of players in a cooperative game, the features derived from REAL biophysical scales should able to collect higher Shapley values (payoffs) with respect to the SHUFFLED ones, which by definition do not convey any biological meaning.

**Figure 2:**
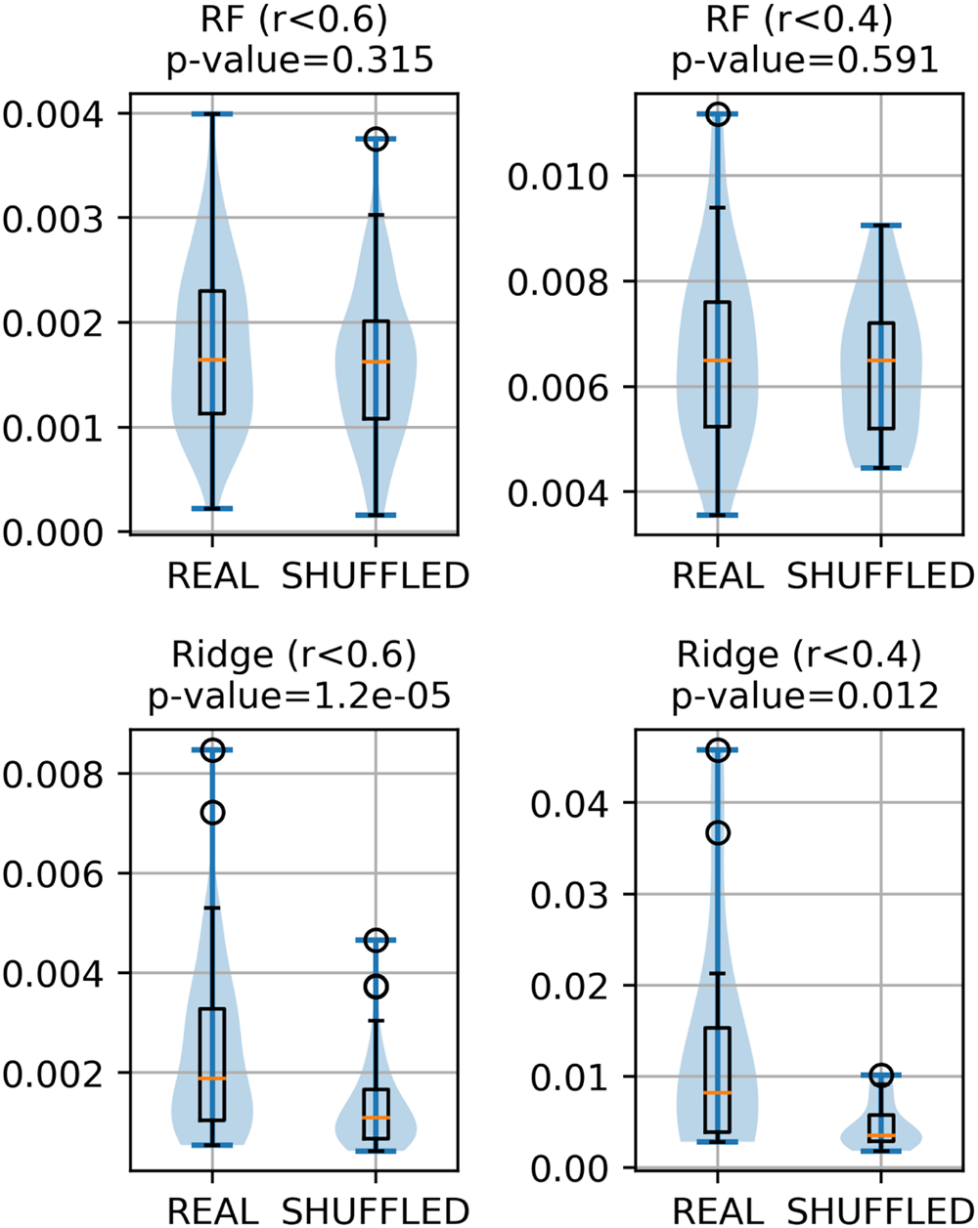
Plots showing the distribution of the approximate Shapley values scores evaluating the contributions of the Real and SHUFFLED features for the RF and Ridge methods on the RSA task. *r* < 0.6 or *r* < 0.4 indicate the maximum Pearson correlation allowed among the pool of sampled scales for each of these experiments. This regulates the maximum allowed scales redundancy used in the experiments.

Figure 2 shows the results of this analysis on the RSA task. The distribution of the Shapley values for the RF method (first row), shows that, regardless of the allowed redundancy of the scales, the p-values are not significant and the distributions are very similar. We thus cannot state that non-linear ML methods can prioritize REAL over SHUFFLED scales when it comes to selecting the most relevant features for the solution of a specific task.

The distributions of the Shapley values for a linear ML method (Ridge)is shown in the second row of Fig. 2. In this case, the Shapley algorithm assigns consistently lower scores to the SHUFFLED scales, and higher scores to the REAL propensity scales. This confirms that, contrary to what could be expected, the use of *biologically meaningful* biophysical propensity scales has very little effect on non-linear ML methods, since these algorithms are *flexible* enough to learn a near-optimal discrimination boundary almost regardless of the encoding used. The same story does not hold for linear methods. The solutions that they can learn has is deemed to be simpler and thus they really benefit from exploiting the information conveyed by a carefully selected feature encoding schemes.

## Discussion

### On the use of propensity scales in bioinformatics

The experiments we performed in this paper show that the efficacy of using propensity scales and different types of ML methods on structural bioinformatics tasks is highly dependent on the problem setting, and that there is no globally optimal solution. In some tasks, linear ML methods with ONEHOT encoding may be optimal, while in other tasks the propensity scales coupled with non-linear ML methods may give the best results. Real propensity scales generally provide significantly better results than randomized scales, but the extent of this advantage is surprisingly small and is often limited to a few percentage points in terms of the best obtainable scores (see Table 1) and negligible effect sizes (<0.3 stds).

This is certainly unexpected and indicates that the widespread use of “biologically meaningful” amino acids descriptions with ML methods may be more likely justified by the fact that it is an intuitive and straightforward solution for the scientists developing the pipeline instead of being an actual way to get better predictions, since highly similar results can be obtained even when their supposed biophysical meaning is removed with randomization. Moreover, when we look at the slightly lower scores obtained with randomized scales, we must also take into account the fact that different types of completely or partially uninformative scales are legit members of the randomized group. For example, scales assigning the same (or extremely similar) values to many amino acid are certainly present among the thousands of randomizations we performed in this study, possibly lowering the final distribution of scores obtained by randomized scales.

### The propensity scales paradox

Our results also highlight a circular behavior, which we call *the propensity scales paradox*. Figure 1 and Table 1 show that the effectiveness of the real propensity scales with respect to the randomized ones varies among the ML methods used, and that this gap is generally greater among linear methods. This means that the use of propensity scales (REAL) coupled with linear methods could be reasonably justified. The paradoxical aspect of this phenomenon is twofold: the first point is that researchers generally prefer to use the better performing non-linear methods to build their models, because these algorithms can learn more complex solutions and thus often give better results. At the same time, the complexity of these models has the side effect of making them able to learn near-optimal solutions almost regardless of the feature encoding that is used, thus defeating the point of using the “biologically meaningful” propensity scales-based feature encoding in the first place.

The second paradoxical aspect is that even among linear methods, when the use of propensity scales could be justified by better performance with respect to the randomized scales, the simplest encoding possible (ONEHOT) shows surprisingly good results. Despite being completely agnostic about the objects that it is describing, the ONEHOT encoding can consistently outperform the biophysical propensity scales. This observation further challenges the usefulness of propensity scales even among linear ML methods, which are more likely to be sensitive to well tuned input encoding.

This success of the ONEHOT encoding may be due to the fact that it uses 20 dimensions to represent each amino acid, therefore allowing even very simple ML methods to precisely assign a weight to the contribution of every type of amino acid in each position of the the sliding windows. On the other hand, the propensity scales allow ML methods to assign a weight to the amino acid biophysical characteristics, which are more general concepts and are thus less specific. If enough scales are used, the ML method may still uniquely identify the amino acids, converging (if necessary) to the same results, but if the scales are not enough or they are not diverse enough, the information provided may not have the necessary specificity to detect subtle amino acid composition differences in the examined sliding windows.

### Why are propensity scales so popular and what are the main drawbacks?

These conceptual issues related to the use of propensity scales coupled with ML methods in structural bioinformatics might have been overlooked until now because of their intuitive appeal. Using values that have a real-world meaning, in this case biophysical, has a strong root in traditional modeling approaches, and might have spared them from a real investigation of their relevance in the modern ML context.

The results of this study indicate that the common practice of starting from a large pool of propensity scales (taken from databases such AAindex) and performing feature selections on them to find the “best” feature encoding to solve a particular structural bioinformatics task it is equivalent to performing a *discrete search for optimal features in a static set of embeddings*, represented by the AAindex scales. In ML, embeddings *e*(*o*_*i*_) are projections of the objects *o*_*i*_∈*O* we want to predict in a space in which the distances *D*(*e*(*o*_*i*_), *e*(*o*_*j*_)) between them reflect characteristics of the objects, such as their (dis)similarity. Embeddings are, for example, widely used in Natural Language Processing, mapping the *sparse* one-hot encoding of words to a *dense* encoding representing *semantic similarities* between them in a generally much lower dimensional space. Optimal embeddings can thus be *learned* by ML methods such as Neural Networks (NN) specifically for the task at hand, performing gradient descent optimization on them similarly to any other weight in the network, in order to extract the optimal feature representation.

Performing a feature selection over the AAindex scales is a way to find an optimal feature representation, but it presents several disadvantages with respect to the embedding-based *feature learning* approach. For example, when using propensity scales, the search is composed by discrete steps (a scale can be added or removed from the set of scales selected so far), and an exhaustive search is computationally intractable, since it requires trying all the possible 2^*N*^ subsets of scales, which is clearly infeasible even for the fastest ML methods. Moreover, the search occurs on a static and finite set of scales, meaning that if the theoretical optimal feature encoding comprises scales which are not present in AAindex, the optimal solution is out of reach. Last, the scales in the AAindex database are heavily redundant between themselves, and indeed only 85 scales over 566 have a Pearson correlation lower than 0.6 between them. This means that even if we consider the feature selection over AAindex scales as a viable feature learning method, AAindex may not be an optimal initial *scales pool* because the high similarity between its elements does not allow a very large exploration of the space of the possible embeddings, and the feature search is doomed to be extremely biased towards the most “popular” amino acid descriptions, thus skewing the *exploitation-exploration* spectrum.

### Which alternatives are available? 

On the other hand, directly learning embeddings would allow a true problem-dependent optimization of the feature encoding using for example a gradient descent framework. Instead of picking amino acid characteristics from a (redundant) set of fixed scales, the ML method can tailor the embedding to represent the most relevant semantic aspects of the encoded objects for the solution of the task at hand. For example, the ML method could infer from the data *and* the task to solve which are the most relevant amino acid characteristics.

To show a practical example of embedding use and to compare them with the scales, we performed an additional experiment. We used a Perceptron, which is a linear NN with no hidden layers, and a non-linear NN with one hidden layer (see Suppl. Mat. for more details) to optimize two sets of six dimensional amino acid embeddings on the SS task. We refer to these embeddings respectively as *e*_*P*_ and *e*_*N*_. We also extracted the six propensity scales used in MAPP^14^, which represent respectively (1) hydropathy, (2) polarity, (3) charge (4) volume (5) free energy in helical and (6) beta-sheet conformation of amino acids. These scale thus include properties that are conceptually important for the SS formation. In the following text we use the notation *M*(*E*) to describe the fact that the ML method *M* has been trained using the feature encoding *E*.

**Figure 3:**
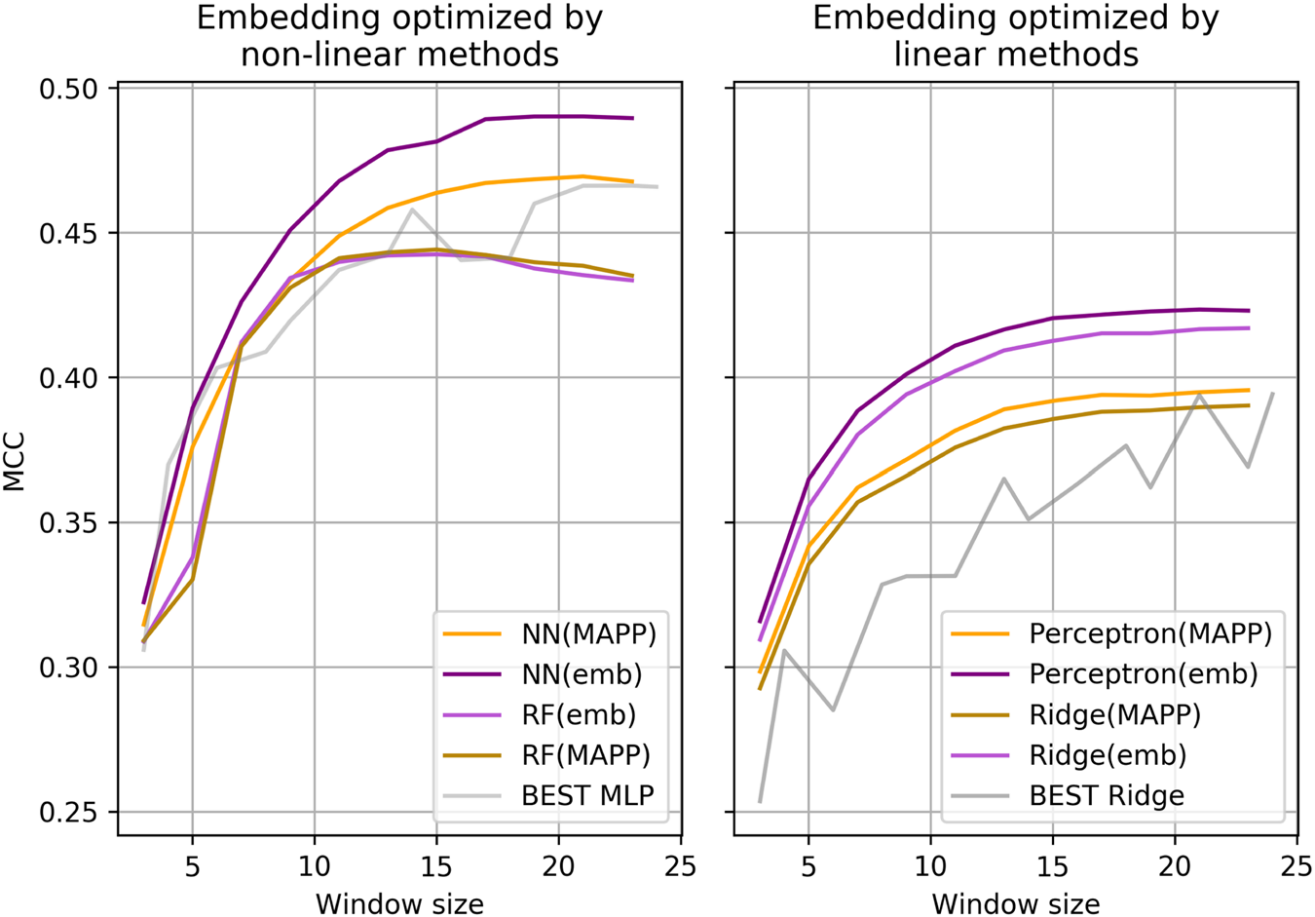
Plot showing the MCC scores obtained by embeddings on non-linear ML methods (left) and linear ones (right). Purple-ish colors indicate experiments using the embeddings, while orange-ish color indicate the MCCs obtained by the same methods but with propensity scales. The grey line represents the best scores for the SS task reported in Table 1).

We then compared the performance of the non-linearly-learned embeddings *e*_*N*_ with the MAPP scales on the RF method and on the native NN that generated *e*_*N*_ on the SS task, separating training and testing data as described in Methods. The left plot in Fig. 3 shows that RF(*e*_*N*_) and RF(MAPP) perform exactly the same, meaning that even transferring *e*_*N*_ from the NN to another method gives scores as good as a comparable set of propensity scales. On the other hand, the NN(*e*_*N*_) outperforms NN(*MAPP*) regardless of the size of the sliding window and outperforms also the MLP score reported in Table 1, which are shown in gray.

In the right plot of Fig. 3, we compare the performance of the embeddings learned from the Perceptron (*e*_*P*_) and the MAPP scales among linear methods. We can clearly see that among linear methods, Ridge(*e*_*P*_) outperforms Ridge(MAPP) and Perceptron(*e*_*P*_) outperforms Perceptron(MAPP) regardless of the number of features used. Moreover, the linear methods using the embedding optimized for the SS task outperform both the best score obtained with Real scales in Table 1 and the 0.40 MCC score obtained by the ONEHOT encoding.

Figure 3 thus shows that problem-specific embeddings can easily outperform propensity scales and that they are *transferable* between ML methods.

## Conclusion

In this paper we reproduce three classical structural bioinformatics prediction problems in controlled experiments to investigate commonly accepted assumptions about ML methods and the optimal encoding of features. In particular, we question the actual usefulness of biophysical propensity scales by comparing their performances with randomized (and thus supposedly *biologically meaningless*) feature encodings across different tasks and ML methods.

It is shown that, although the widely used propensity scales obtain slightly higher performances, extremely similar results can be obtained by removing their supposed biophysical meaning by shuffling or randomly generating scales. Moreover, the results indicate that the effectiveness of the real propensity scales varies among the ML methods used, and while it is greater with linear methods, researchers generally prefer the more performant non-linear methods, in which the specific biophysical encoding is even less relevant. Even in the case of linear ML methods, which are likely to benefit from the scales, the simplest possible encoding, called ONEHOT, can outperform the biologically meaningful scales, as was also initially hypothesized in one of the first structural bioinformatics studies^40^.

Last, results show that non-linear ML methods may not be able to distinguish between randomized and real propensity scales by assigning more relevance to the latter in a feature selection settings, and we conclude by suggesting that learning embeddings could be a simple, assumptions-free and optimal way to perform feature learning/engineering in structural bioinformatics tasks.

## Supplementary information

Supplementary Material

## Data Availability

All the data and the code are publicly available.
