# Supplementary material for: Exploring the limitations of biophysical propensity scales coupled with machine learning for protein sequence analysis
Source: Sci Rep. 2019 Nov 15;9:16932. doi: 10.1038/s41598-019-53324-w (PMC6858301; doi:10.1038/s41598-019-53324-w)
Supplement: Supplementary file 1 — Supplementary Material [file 41598_2019_53324_MOESM1_ESM.pdf]

Exploring the limitations of biophysical propensity scales coupled with machine learning for protein sequence analysis
(Supplementary Material)

D. Raimondi, G. Orlando, W. Vranken, Y. Moreau

September 26, 2019

S1 Datasets

The SCRATCH-1D dataset is available from: http://download.igb.uci.edu/ .

PDBCYS dataset is available from: http://www.biocomp.unibo.it/savojard/PDBCYS.ssbonds.txt .

S2 ML methods hyper-parameters

The parameters used by the MLP, RF, Ridge and LinSVC were the default ones provided by the scikit-learn implementation, except for the RF, which uses 50 trees and the MLP, which has 50 hidden units, a maximum number of epochs set to 70.

We list the full sets of parameters here.

```
sklearn.neural_network.MLPClassifier(hidden_layer_sizes=(50, ), activation="relu",
solver="adam", alpha=0.0001, batch_size="auto",
learning_rate="constant", learning_rate_init=0.001, power_t=0.5, max_iter=70,
shuffle=True, random_state=None, tol=0.0001, verbose=False, warm_start=False,
momentum=0.9, nesterovs_momentum=True, early_stopping=False, validation_fraction=0.1,
beta_1=0.9, beta_2=0.999, epsilon=1e-08, n_iter_no_change=10)
```

```
sklearn.ensemble.RandomForestClassifier(n_estimators=50, criterion="gini",
max_depth=None, min_samples_split=2, min_samples_leaf=1, min_weight_fraction_leaf=0.0,
max_features="auto", max_leaf_nodes=None, min_impurity_decrease=0.0,
min_impurity_split=None, bootstrap=True, oob_score=False,
n_jobs=None, random_state=None, verbose=0, warm_start=False, class_weight=None)
```

```
sklearn.linear_model.RidgeClassifier(alpha=1.0, fit_intercept=True,
normalize=False, copy_X=True, max_iter=None, tol=0.001, class_weight=None,
solver="auto", random_state=None)
```

```
sklearn.svm.LinearSVC(penalty="l2", loss="squared_hinge", dual=True, tol=0.0001,
C=1.0, multi_class="ovr", fit_intercept=True, intercept_scaling=1, class_weight=None,
verbose=0, random_state=None, max_iter=1000)
```

S3 Evaluation metrics

The Matthews Correlation Coefficient used to evaluate the SS prediction task is computed as follows:

$$MCC = \frac{TP \times TN - FP \times FN}{\sqrt{(TP + FP)(TP + FN)(TN + FP)(TN + FN)}}$$

and

$$F1 = \frac{2TP}{2TP + FP + FN}$$

where TP, TN, FP and FN are respectively the true positives, true negatives, false positives and false negatives.

The Cohen's d effect size has been computed as follows:

$$d = \frac{\mu_1 - \mu_2}{s} \qquad s = \sqrt{\frac{(n_1 - 1)\sigma_1^2 + (n_2 - 1)\sigma_2^2}{n_1 + n_2 - 2}}$$

where μ_i is the mean of the sample i, s is the pooled standard deviation and σ^2 is the unbiased estimator of the variance.

S4 Performance in function of the feature size

Fig. S1 shows the performance of the ML methods on the 3 structural bioinformatics tasks in function of the number of features used. The lines represent the mean scores and the error bars indicate the standard deviation within each of the 23 bins used.

Overall, we can see that there is a clear positive correlation between the AUC (MCC in the case of the SS prediction) scores obtained and the number of features used, regardless of the feature encoding. In particular, we can see that the Shuffled and Random scores (respectively in yellow and green) are generally superimposed, while the use of Real scales yield to higher means. Nonetheless, these means fall very often within the error bars of the scores obtained by the randomized experiments.

In the RSA and SS experiments, non-linear models tend to reach optimal or near-optimal scores with relatively small feature sizes (between 50 and 100 dimensions) and show little benefit when this number is increased. On the other hand, linear models scores are generally more scattered, in particular in the LinSVC case. A striking point concerning linear models is that in the SS and RSA experiments, ONEHOT encoding consistently outperforms the other encodings, regardless of the number of features used. This is not the case for the CYS prediction, where ONEHOT performs poorly regardless of the ML method used.

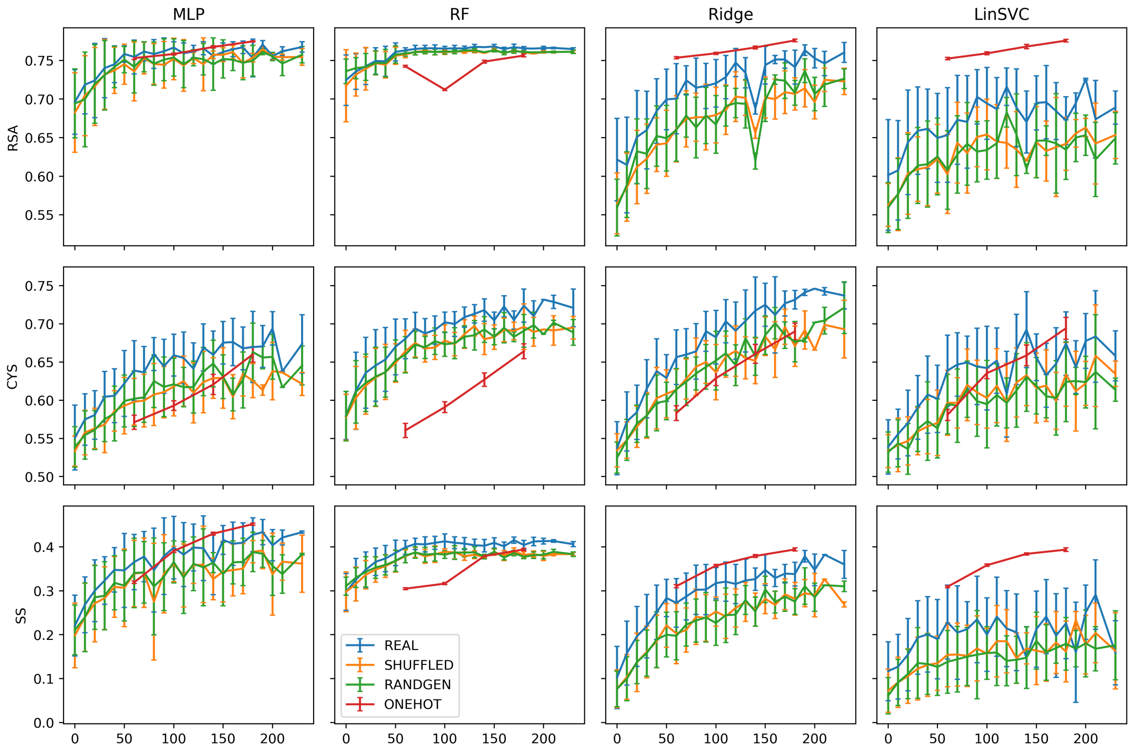

Figure S1: Performances of the four ML methods on the 3 tasks tested in this study in function of the number of features used during each of the randomizations shown in Fig. 1. The lines have been obtained by binning the x axis in 23 bins of size 10 and plotting the mean scores obtained within each bin. The error bars indicate the standard deviation in each bin.

S5 Description of the NN and Perceptron models

Here we provide the detailed description of the Neural Network (NN) and Perceptron models used in Section 4.4 of the main paper.

We built both models using pytorch version 0.3.1 (https://pytorch.org/).

The Perceptron is has no hidden layers, and produces 3 outputs with a Softmax activation, since the SS prediction is multiclass. The NN has one hidden layer with 50 neurons with ReLU activations. The output layer has the same 3 neurons as the Perceptron.

For both the models, the windows size is fixed to 17 residues. The only difference between the NN and Perceptron using embeddings instead of MAPP scales is that the embeddings are defined as `self.e = t.nn.Embedding(21,6)` . They have the same number of dimensions of the MAPP scales. The 21st dimensions is used to represent the padding fo the incomplete sliding windows (the one at the beginning and at the end of the proteins). In the MAPP case, we used zero padding, similarly to all the other experiments presented in the paper.

Listing 1: Perceptron(MAPP) pytorch code

```
class Perceptron(t.nn.Module):

        def __init__(self,  WS):
                super(Perceptron, self).__init__()
                # WS is the window size. we used WS=17
                self.f = t.nn.Sequential( t.nn.Linear(WS*6, 3), t.nn.Softmax())
                #the softmax provide 3D output

        def forward(self, x):
                o = self.f(x)
                return o
                }
```

Listing 2: NN(MAPP) pytorch code

```
class NN(t.nn.Module):

        def __init__(self,  WS):
                super(NN, self).__init__()
                # WS is the window size. we used WS=17
                self.f = t.nn.Sequential( t.nn.Linear(WS*6,50), t.nn.ReLU(),\
                 t.nn.Linear(50,3), t.nn.Softmax())
                #the softmax provide 3D output

        def forward(self, x):
                o = self.f(x)
                return o
                }
```

Listing 3: NN(emb) pytorch code

```
class NN(t.nn.Module):

        def __init__(self,  WS):
                super(NN, self).__init__()
                # WS is the window size. we used WS=17
                self.e = t.nn.Embedding(21,6)
                self.f = t.nn.Sequential( t.nn.Linear(WS*6,50), t.nn.ReLU(),\
                 t.nn.Linear(50,3), t.nn.Softmax())
                #the softmax provide 3D output

        def forward(self, x):
                e1 = self.e(x).view(x.size(0), x.size(1)*6)
                o = self.f(e1)
                return o
                }
```

Listing 4: Perceptron(emb) pytorch code

```
class Perceptron(t.nn.Module):

        def __init__(self,  WS):
                super(Perceptron, self).__init__()
                # WS is the window size. we used WS=17
                self.e = t.nn.Embedding(21,6)
                self.f = t.nn.Sequential( t.nn.Linear(WS*6,3), t.nn.Softmax())
                #the softmax provide 3D output

        def forward(self, x):
                e1 = self.e(x).view(x.size(0), x.size(1)*6)
                o = self.f(e1)
                return o
                }
```

We used the Cross Entropy loss function. For the training, we used the Adam optimizer starting with a learning rate of 1e-2. We scheduled a 0.5 reduction of the learning rate every 5 epochs with no improvement of the training error. All these parameters are shown below.

Listing 5: Perceptron(emb) pytorch code

```
        loss_function = t.nn.CrossEntropyLoss(size_average=False)

        #######OPTIMIZER#########
        self.learning_rate = 1e-2
        optimizer = t.optim.Adam(self.model.parameters(), lr=self.learning_rate,\
         weight_decay=0)
        scheduler = t.optim.lr_scheduler.ReduceLROnPlateau(optimizer, \
         mode='min', factor=0.5, patience=5, verbose=True, \
         threshold=0.0001, threshold_mode='rel', cooldown=0, min_lr=0, eps=1e-08)
```

S6 Analysis of the embeddings learned

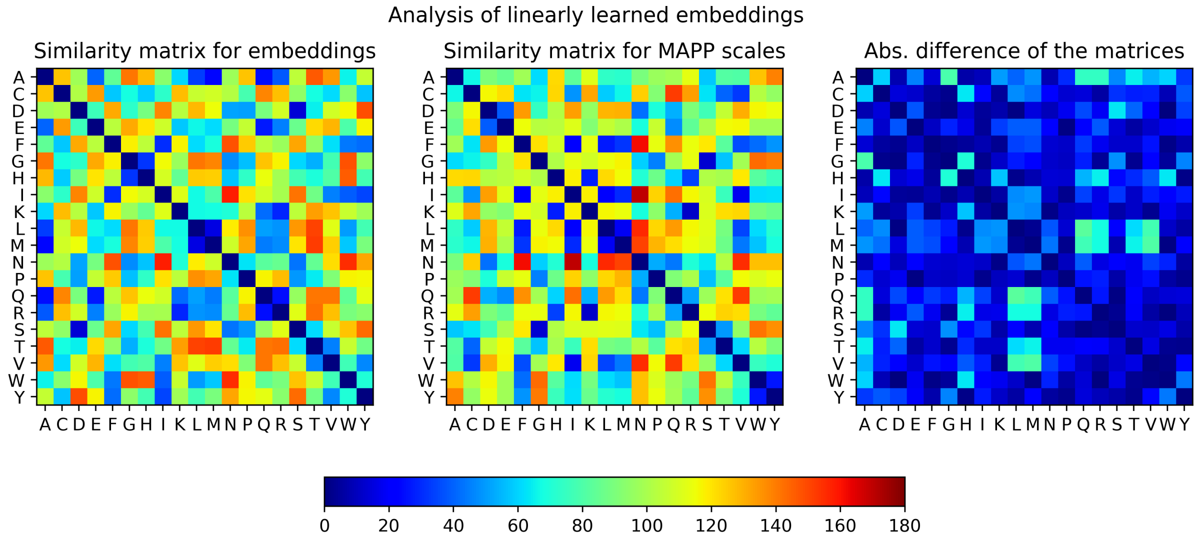

Figure S2: Figure showing the comparison between the embeddings learned by the Perceptron on the SS task (left plot) with the MAPP scales (central plot). The right plot shows the difference between the two previous matrices. The values represented are the angles between the vectors, measured in degrees.

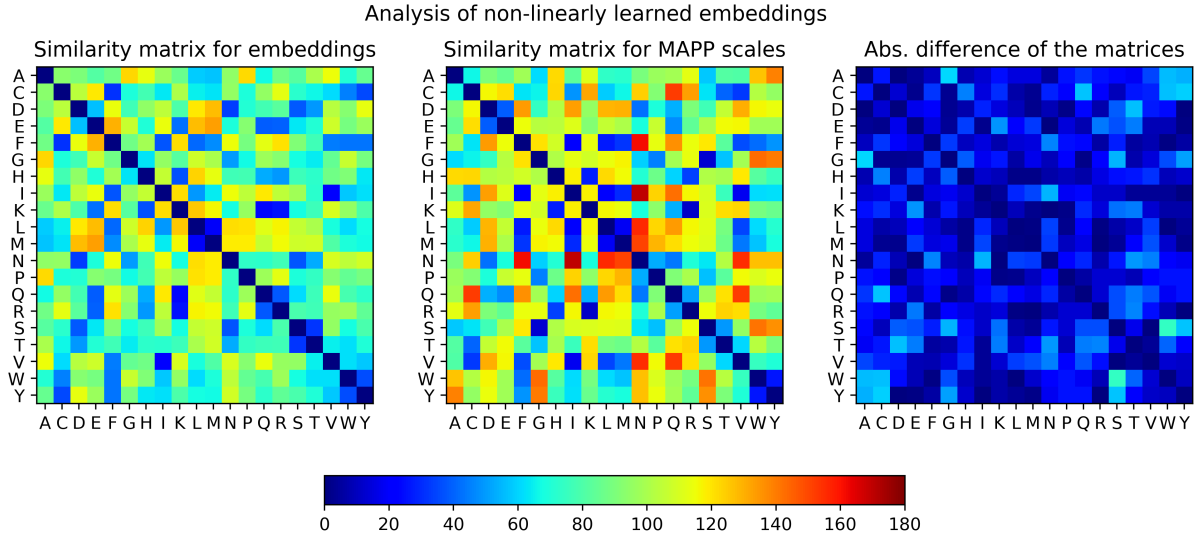

Figure S3: Figure showing the comparison between the embeddings learned by the Neural Network on the SS task (left plot) with the MAPP scales (central plot). The right plot shows the difference between the two previous matrices. The values represented are the angles between the vectors, measured in degrees.

S7 Randomization experiment on the homology detection task

Besides the randomization experiments on the SS, RSA and OXCYS tasks, presented in the main manuscript, we ran an additional shorter experiment showing that the same behavior occurs when coupling biophysical propensity scales and non-linear machine learning methods to tackle the protein homology detection task. To do so we adapted a previously developed and recently published in-house alignment-free global homology detection method, called WARP [1].

WARP [1] is an alignment-free homology detection method which:

1. takes pairs of proteins,
2. describes their sequence with a certain number of biophysical characteristics,
3. compresses these description using the inverse Discrete Cosine Transform,
4. computes the similarity between the compressed profile corresponding to each feature type with the Dynamic Time Warping algorithm and
5. uses these similarity values as input feature vectors in a Random Forest predictor trained to distinguish between homologous (similar proteins) from non-homologous proteins.

For more details about the mehtod, please refer to [1]. In the original publication the biophysical characteristics used to numerically describe protein sequences in WARP were the PSIPRED Secondary Structure [2] assignments and the protein backbone dynamics predictions obtained with DynaMine [3], plus the 1-hot encoding representation of the protein sequence.

For this study, we adapted WARP code to use biophysical propensity scales taken from AAindex as biophysical description of the protein sequences. Similarly to the SS, RSA and OXCYS experiments, we reduced the redundancy of the AAindex scales by allowing no more than 0.6 of Pearson correlation between the selected scales, obtaining a pool of 85 propensity scales. We ran 100 training/testing iterations on WARP over the PFAM dataset of homologous protein pairs, which contains 5245 homologous protein pairs and 5245 non-homologous pairs (see [1] for more details). In each iteration we randomly selected 5 propensity scales from the 85 scales pool, and we trained and tested WARP two times: the first while using the sampled scales (REAL scales), and the second after a random shuffling of the same scales (SHUFFLED scales). We thus collected 100 AUC values describing the performances of WARP when describing protein sequences with the REAL propensity scales and 100 AUC values obtained after shuffling the same scales.

In Fig. S4 we show the distributions of the AUCs for the REAL and SHUFFLED scales obtained in this 100 iterations experiments. From the plot we can see that even if we use SHUFFLED (and thus devoid of biological meaning) propensity scales to describe protein sequences within the WARP pipeline, the performances obtained are i) significantly better than random and ii) relatively similar to the scores obtained with REAL propensity scales.

Moreover, in Table S1 we show the best AUC and AUPRC values obtained among all the iterations, both with REAL and SHUFFLED scales, and we can see that, although AUPRC of the REAL scales is 9% higher, the AUC scores are nearly equivalent.

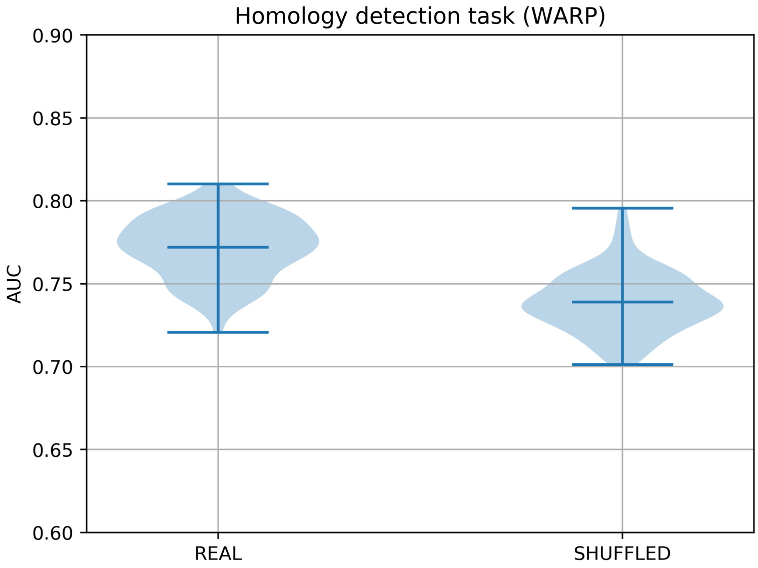

Figure S4: Figure showing distributions of the AUC scores obtained by WARP model [1] in the homology detection task on the PFAM dataset (see [1] for details). The distribution on the left (REAL) corresponds to the AUC scores obtained by randomly selecting 5 scales from a non-redundant subset of AAindex, while the one one on the right corresponds to the AUC scores obtained after randomly shuffling the same 5 scales. The distributions are composed by 100 iterations of this procedure.

Scores	REAL	SHUFFLED
AUC	0.81	0.80
AUPRC	0.87	0.8

Table S1: Table reporting the best AUC and AUPRC scores obtained in the homology detection experiment

References

[1] Raimondi D, Orlando G, Moreau Y, Vranken WF. Ultra-fast global homology detection with Discrete Cosine Transform and Dynamic Time Warping. Bioinformatics. 2018;1:8.

[2] Buchan DW, Minneci F, Nugent TC, Bryson K, Jones DT. Scalable web services for the PSIPRED Protein Analysis Workbench. Nucleic acids research. 2013;41(W1):W349–W357.

[3] Cilia E, Pancsa R, Tompa P, Lenaerts T, Vranken WF. From protein sequence to dynamics and disorder with DynaMine. Nature communications. 2013;4.
